# Serum Potassium Levels and Mortality in Hospitalized Heart Failure Patients

**DOI:** 10.31083/j.rcm2408228

**Published:** 2023-08-09

**Authors:** Bo-Ping Huang, Lang Zhao, Xue-Mei Zhao, Mei Zhai, Yan Huang, Qiong Zhou, Peng-Chao Tian, Lin Liang, Li-Yan Huang, Jia-Yu Feng, Yu-Hui Zhang, Jian Zhang

**Affiliations:** ^1^Heart Failure Center, State Key Laboratory of Cardiovascular Disease, Fuwai Hospital, National Center for Cardiovascular Diseases, Chinese Academy of Medical Sciences & Peking Union Medical College (CAMS & PUMC), 100037 Beijing, China; ^2^Key Laboratory of Clinical Research for Cardiovascular Medications, National Health Committee, 100037 Beijing, China

**Keywords:** serum potassium, heart failure, outcome, hypokalemia, hyperkalemia

## Abstract

**Background:**

To assess the link between serum potassium ($K^{+}$) and all-cause mortality in hospitalized heart failure (HF) patients.

**Methods:**

Hospitalized HF patients (n = 3114) were analyzed at the Fuwai Hospital Heart Failure Center. Before discharge, HF patients were divided into four groups according to the $K^{+}$ level quartiles: $K^{+}$ ≤3.96 mmol/L (Q1), 3.96 < $K^{+}$ ≤ 4.22 mmol/L (Q2), 4.22 < $K^{+}$ ≤ 4.52 mmol/L (Q3), and $K^{+}$ >4.52 mmol/L (Q4). At 90 days, 2 years, and maximal follow-up, all-cause mortality was the primary outcome.

**Results:**

Patients with HF in the Q4 group had worse cardiac function, higher N-terminal pro-B-type natriuretic peptide levels, lower left ventricular ejection fractions and lower estimated glomerular filtration rates than patients in the Q2 group. In the multivariate-adjusted Cox analysis, the mortality assessed during the 90-day, 2-year, and maximal follow-up examinations increased in the Q4 group of HF patients but not in the Q1 and Q3 groups. The Q4 group had a 28% (hazard ratio [HR]: 1.28, 95% confidence interval [CI]: 1.09–1.49, *p* = 0.002) higher risk of all-cause mortality at maximum follow-up. Hypokalemia and hyperkalemia were linked to increased HF mortality risk at the 90-day, 2-year, and maximal follow-up periods.

**Conclusions:**

Serum $K^{+}$ levels had a J-shaped association with all-cause mortality in HF patients. Both hypokalemia and a $K^{+}$ level of >4.52 mmol/L were associated with increased all-cause mortality in the short term and long term, suggesting a narrow target $K^{+}$ range in HF patients.

**Clinical Trial Registration:**

Unique Identifier: NCT02664818; URL: clinicaltrials.gov

## 1. Introduction

Heart failure (HF) is becoming more common, and the associated mortality and morbidity rates remain high [1, 2]. Guidelines recommend diuretics, angiotensin II receptor blockers (ARBs) or angiotensin-converting enzyme inhibitors (ACEIs), and mineralocorticoid receptor antagonists (MRAs) for HF patients, but these drug treatments may contribute to dyskalemia [3, 4, 5]. The comorbidities and pathophysiology of HF further increase the risk for dyskalemia [6, 7]. Hypokalemia and hyperkalemia are usually defined as serum potassium ($K^{+}$) concentrations below and above 3.5–5.0 mmol/L, respectively. A U-shaped relationship between $K^{+}$ levels and mortality in acute myocardial infarction, hypertension, chronic HF, and acute HF following myocardial infarction has been reported; lower and higher $K^{+}$ levels in the normal range and hyperkalemia are linked to higher short-term mortality [8, 9, 10, 11, 12]. It is unclear whether these results also apply to hospitalized HF patients. The clinical features of serum $K^{+}$ levels in hospitalized HF patients and the association between serum $K^{+}$ levels and poor clinical outcomes have not been well characterized. We examined the distribution of $K^{+}$ levels, their connection to clinical features, and the relationship between serum $K^{+}$ concentrations and 90-day, 2-year, and maximal follow-up all-cause mortality in hospitalized HF patients.

## 2. Materials and Methods

### 2.1 Participants

This retrospective analysis of the prospective cohort study was performed at the HF Center of Fuwai Hospital between December 2006 and December 2017. The patients, including chronic decompensated HF and new-onset HF patients, were continuously enrolled. The diagnosis and assessment of hospitalized HF patients were based on symptoms/signs of fluid overload or hypoperfusion and relevant laboratory, functional, and imaging tests (such as measurements of N-terminal pro-B-type natriuretic peptide (NT-proBNP), echocardiography, electrocardiogram, and chest X-ray). The inclusion criteria were as follows: at least one of the signs and symptoms of HF; New York Heart Association (NYHA) class Ⅱ–Ⅳ; and NT-proBNP levels >300 pg/mL.

We did not include patients with missing serum $K^{+}$ level information or missing follow-up data. Patients without outcome event times were excluded. In addition, those who died while hospitalized were eliminated. This study included 3114 hospitalized patients with HF in the analysis (**Supplementary Fig. 1**). Serum potassium values were measured in all patients from 2 days before discharge to the day of discharge. The institutional ethics committee of Fuwai Hospital authorized the study protocol after verifying that it adhered to the principles of the Helsinki Declaration. The patients signed individual informed consent forms.

### 2.2 Potassium Intervals

The patients in the study were categorized based on quartiles of serum $K^{+}$ levels before discharge: $K^{+}$ ≤3.96 mmol/L (Q1), 3.96 < $K^{+}$ ≤ 4.22 mmol/L (Q2), 4.22 < $K^{+}$ ≤ 4.52 mmol/L (Q3), and $K^{+}$ >4.52 mmol/L (Q4). The serum $K^{+}$ range of the Q2 group was a reference for statistical analysis. The normal $K^{+}$ range was 3.5–5.0 mmol/L. Hypokalemia and hyperkalemia were considered $K^{+}$ levels of <3.5 mmol/L and >5.0 mmol/L, respectively.

### 2.3 Baseline Study Variables

Clinical details regarding demographics, comorbidities, blood biochemistry results, echocardiograms, and medication data were recorded before discharge. The use of ACEIs or ARBs, β-blockers, MRAs, digoxin, thiazides, loop diuretics, and other diuretic drugs was assessed. To assess renal function in HF patients, the estimated glomerular filtration rate (eGFR) was employed [13, 14]. Age, sex, and serum creatinine level at baseline were utilized to assess renal function status.

### 2.4 Follow-Up and Outcomes

Patients regularly attended followed-up appointments at the outpatient clinic and by telephone after discharge until January 2020, at least once every 3 months within the first year, and every 6 months thereafter. Patients were followed up until cardiovascular or all-cause death occurred. The medical records of patients who were followed up in the Fuwai hospital system provided information on occurrences of adverse events. For patients who were not followed up in our hospital, if necessary, the patient’s relatives and local medical personnel were contacted by telephone to obtain detailed information. Two blinded cardiologists examined and analyzed adverse event data. At 90-days, 2-years, and the maximal follow-up, the primary outcome was all-cause death. The survival duration was computed from the discharge date to the death or final follow-up date.

### 2.5 Statistical Analysis

This study included four $K^{+}$ intervals, with the reference interval selected as 3.96 < $K^{+}$ ≤ 4.22 mmol/L (Q2). Continuous (mean ± SD or median) and categorical (counts and percentages) variables are displayed in the baseline table. Pearson chi-square (proportions) and ANOVA (continuous variables) were used to compare baseline variables across patients with various $K^{+}$ levels. In addition, the Kruskal‒Wallis rank test was used to assess nonnormally distributed continuous variables. Kaplan-Meier cumulative mortality curves are shown for the quartiles of the serum $K^{+}$ intervals and illustrate the trend of mortality over time. Clinical comorbidities, laboratory parameters, and important cardiovascular medications were considered covariates in the analysis. A Cox regression model was applied to evaluate the relationship between serum $K^{+}$ levels and mortality within 90 days, 2 years, and maximal follow-up after adjusting for the defined covariates. The proportional hazard assumption was fulfilled by the Cox regression model. The adjusted variables were chosen based on clinical knowledge, the findings of univariate analyses, and their potential relevance to hypokalemia or hyperkalemia and/or outcomes. Moreover, restricted cubic splines were employed to examine the link between serum $K^{+}$ levels and all-cause mortality. In a two-sided test, *p* < 0.05 was considered to indicate statistical significance. Multiple imputation statistical methods were used to address missing data. R version 4.0.3 (R Foundation for Statistical Computing, Vienna, Austria) was used for the analysis.

## 3. Results

### 3.1 Participant Characteristics

This analysis enrolled 3114 hospitalized HF patients in total, with a mean follow-up of 4.14 years. The majority of HF patients (93.3%) had normal $K^{+}$ values (3.5–5.0 mmol/L), whereas 52 (1.7%) and 158 (5.1%) patients had hypokalemia and hyperkalemia, respectively. On admission, the average $K^{+}$ value was 4.02 ± 0.51 mmol/L, and it was 4.27 ± 0.45 mmol/L before discharge. **Supplementary Fig. 2** shows the distribution of $K^{+}$ levels before discharge. The patient characteristics based on the quartiles of serum $K^{+}$ levels before discharge are summarized in Table 1. The mean age was 56.93 ± 16.04 years, and 2208 patients (70.9%) were male. In addition, 1496 (48.0%) and 1038 (33.3%) had a history of hypertension and diabetes, respectively. Among patients with various serum $K^{+}$ levels, there was no statistically significant distinction in the usage of drugs (digoxin, ACEIs or ARBs, beta-blockers, MRAs, and diuretics) compared to the Q2 group.

**Table 1.** **Baseline features of heart failure patients based on serum potassium quartiles**.

Parameters		Total	$K^{+}$ ≤3.96 mmol/L	3.96 < $K^{+}$ ≤ 4.22 mmol/L	4.22 < $K^{+}$ ≤ 4.52 mmol/L	$K^{+}$ >4.52 mmol/L	*p* value
		(n = 3114)	(Q1, n = 780)	(Q2, n = 791)	(Q3, n = 770)	(Q4, n = 773)	
Age (years)		56.93 ± 16.04	53.81 ± 16.52	55.57 ± 15.28	57.89 ± 15.92	60.51 ± 15.68	<0.001
Male, n (%)		2208 (70.9)	570 (73.1)	582 (73.6)	521 (67.7)	535 (69.2)	0.023
Body mass index (kg/$m^{2}$)		24.45 ± 4.36	24.84 ± 4.51	24.82 ± 4.62	24.05 ± 4.06	24.07 ± 4.14	<0.001
Heart rate (bpm)		81.13 ± 18.45	81.41 ± 18.46	81.18 ± 18.18	81.58 ± 18.60	80.34 ± 18.57	0.556
Coronary artery disease, n (%)		1218 (39.1)	287 (36.8)	283 (35.8)	294 (38.2)	354 (45.8)	<0.001
Hypertension, n (%)		1496 (48.0)	356 (45.6)	372 (47.0)	338 (43.9)	430 (55.6)	<0.001
Diabetes, n (%)		1038 (33.3)	237 (30.4)	262 (33.1)	259 (33.6)	280 (36.2)	0.111
Systolic blood pressure (mmHg)		118.45 ± 20.26	117.36 ± 20.15	118.44 ± 20.87	118.30 ± 19.91	119.70 ± 20.07	0.154
Diastolic blood pressure (mmHg)		71.67 ± 13.28	71.63 ± 13.45	71.96 ± 13.70	71.59 ± 12.93	71.48 ± 13.05	0.901
NYHA class, n (%)							<0.001
	II	721 (23.2)	211 (27.1)	205 (25.9)	139 (18.1)	166 (21.5)	
	III	1532 (49.2)	380 (48.7)	370 (46.8)	423 (54.9)	359 (46.4)	
	IV	861 (27.6)	189 (24.2)	216 (27.3)	208 (27.0)	248 (32.1)	
Hemoglobin (g/L)		136.84 ± 23.30	138.59 ± 23.01	138.85 ± 22.56	136.18 ± 23.57	133.68 ± 23.72	<0.001
Total protein (g/L)		68.25 ± 7.34	67.90 ± 7.33	68.45 ± 7.05	68.59 ± 7.59	68.06 ± 7.38	0.203
Albumin (g/L)		39.49 ± 5.34	39.89 ± 5.48	40.07 ± 5.12	39.60 ± 5.19	38.39 ± 5.41	<0.001
ALT (IU/L)		22.00 [14.00, 37.00]	24.00 [16.00, 37.00]	23.00 [15.00, 38.00]	21.00 [14.25, 36.00]	20.00 [13.00, 36.00]	0.004
AST (IU/L)		24.00 [19.00, 33.00]	23.00 [18.00, 32.00]	24.00 [18.00, 33.00]	24.00 [19.00, 33.00]	24.00 [19.00, 33.00]	0.478
Total bilirubin (μmol/L)		20.70 [14.50, 31.60]	19.75 [14.50, 29.30]	21.30 [14.80, 32.65]	21.45 [14.60, 32.00]	20.60 [14.30, 32.20]	0.073
Direct bilirubin (μmol/L)		4.20 [2.70, 7.30]	3.90 [2.60, 6.53]	4.30 [2.70, 7.40]	4.40 [2.80, 7.40]	4.50 [2.80, 8.40]	0.004
Na (mmol/L)		137.07 ± 4.43	137.44 ± 4.25	137.35 ± 4.21	136.86 ± 4.39	136.60 ± 4.82	<0.001
eGFR (mL/min/1.73 $m^{2}$)		70.81 ± 29.33	76.80 ± 27.62	76.21 ± 29.01	70.37 ± 28.81	59.67 ± 28.63	<0.001
Triglyceride (mmol/L)		1.32 [0.98, 1.82]	1.38 [1.01, 1.87]	1.32 [0.99, 1.83]	1.32 [0.98, 1.79]	1.28 [0.96, 1.79]	0.065
Total cholesterol (mmol/L)		4.17 ± 1.18	4.23 ± 1.26	4.12 ± 1.09	4.22 ± 1.20	4.09 ± 1.16	0.050
High-density lipoprotein (mmol/L)		0.99 ± 0.31	1.00 ± 0.30	0.98 ± 0.29	0.99 ± 0.31	0.99 ± 0.33	0.428
Low-density lipoprotein (mmol/L)		2.56 ± 0.92	2.61 ± 1.01	2.53 ± 0.86	2.59 ± 0.90	2.49 ± 0.93	0.054
C-reactive protein (mg/L)		4.83 [2.55, 11.10]	4.18 [2.14, 8.56]	4.36 [2.43, 9.93]	5.19 [2.76, 12.20]	5.68 [3.01, 14.40]	<0.001
BUN (mmol/L)		8.78 ± 4.51	8.32 ± 4.57	8.15 ± 3.79	8.90 ± 4.27	9.77 ± 5.16	<0.001
Uric acid (μmol/L)		466.67 ± 161.67	462.67 ± 161.91	462.71 ± 155.52	465.25 ± 163.47	476.17 ± 165.70	0.298
HSCRP (mg/L)		3.72 [1.68, 10.43]	3.02 [1.33, 8.46]	3.37 [1.54, 9.54]	4.27 [1.80, 10.98]	4.80 [2.20, 11.48]	<0.001
NT-proBNP (pg/mL)		2207.5 [1023.3,4781.8]	1774.5 [923.8, 4060.8]	2086.0 [914.0, 4167.0]	2235.5 [1097.3, 4934.5]	2796.0 [1197.0, 5861.0]	<0.001
LVEF, n (%)							0.133
	<40	1733 (55.7)	408 (52.3)	459 (58.0)	429 (55.7)	437 (56.5)	
	≥40	1381 (44.3)	372 (47.7)	332 (42.0)	341 (44.3)	336 (43.5)	
Pharmacotherapy							
Digoxin, n (%)		1749 (56.2)	425 (54.5)	465 (58.8)	445 (57.8)	414 (53.6)	0.109
ACEIs/ARBs, n (%)		2335 (75.0)	576 (73.8)	579 (73.2)	598 (77.7)	582 (75.3)	0.182
Beta-blocker, n (%)		2657 (85.3)	677 (86.8)	656 (82.9)	656 (85.2)	668 (86.4)	0.127
MRAs, n (%)		2111 (67.8)	541 (69.4)	519 (65.6)	510 (66.2)	541 (70.0)	0.161
Thiazides, n (%)		154 (4.3)	44 (4.8)	36 (3.9)	38 (4.5)	36 (4.0)	0.791
Loop diuretics, n (%)		2452 (78.7)	602 (77.2)	611 (77.2)	618 (80.3)	621 (80.3)	0.218
Diuretic, n (%)		3001 (96.4)	743 (95.3)	758 (95.8)	748 (97.1)	752 (97.3)	0.086

ACEIs, angiotensin-converting enzyme inhibitors; AST, aspartate aminotransferase; BUN, blood urea nitrogen; NYHA, New York Heart Association; ALT, alanine transaminase; LVEF, left ventricular ejection fraction; eGFR, estimated glomerular filtration rate; NT-proBNP, N-terminal pro-B-type natriuretic peptide; ARBs, angiotensin II receptor blockers; MRAs, mineralocorticoid receptor antagonists; HSCRP, high-sensitivity C-reactive protein.

Patients in the Q4 group had a higher proportion of coronary heart disease, hypertension, diabetes, and NYHA class IV than patients in the Q2 group. Patients in the Q4 group had lower levels of hemoglobin, albumin, and eGFR but higher NT-proBNP and high-sensitivity C-reactive protein and a higher age than patients in all other groups.

### 3.2 Association between Serum Potassium Level and Outcome

**Fig. 1.**
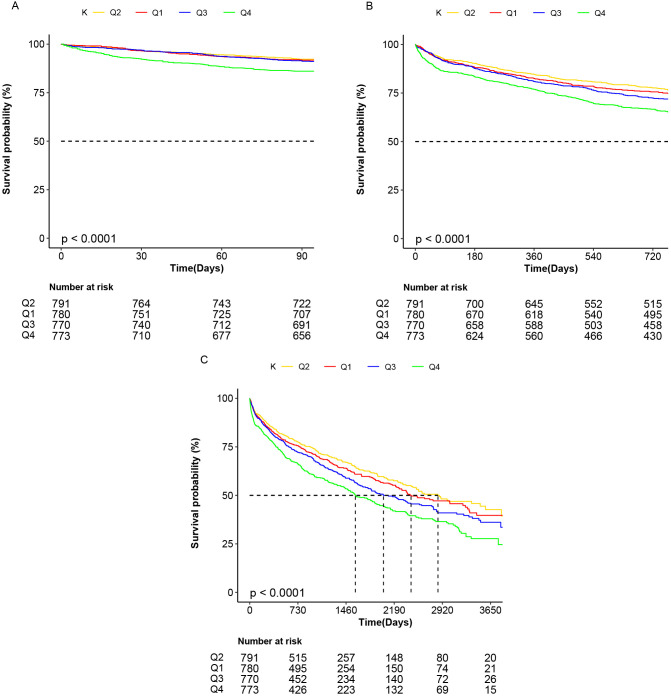
**Kaplan-Meier curve of survival probability of patients based on serum potassium quartiles**. Ninety-day (A), 2-year (B), and maximal (C) follow-up survival by four groups: red, $K^{+}$ ≤3.96 mmol/L (Q1); yellow, 3.96 < $K^{+}$ ≤ 4.22 mmol/L (Q2); blue, 4.22 < $K^{+}$ ≤ 4.52 mmol/L (Q3), and green, $K^{+}$ >4.52 mmol/L (Q4).

A total of 1300 deaths (41.7%) occurred during follow-up. The 2-year mortality rates in the quartiles of the serum $K^{+}$ levels from the lowest ($K^{+}$ ≤3.96 mmol/L, Q1) to the highest ($K^{+}$ >4.52 mmol/L, Q4) were 23.3%, 21.2%, 26.1%, and 32.0%, respectively. At the 90-day, 2-year, and maximal follow-up, the crude survival rate of patients in the Q4 group was the worst (Fig. 1). At 2 years, patients in the Q4 group had worse survival than those for all other groups, whereas those for the Q1 and Q3 groups were comparable. The Q2 group patients had a greater 2-year survival rate than the other groups.

Those with hypokalemia ($K^{+}$ <3.5 mmol/L) and hyperkalemia ($K^{+}$ >5.0 mmol/L) had a substantially greater risk of all-cause mortality than those in the Q2 group. Patients with $K^{+}$ levels >5.0 mmol/L had the lowest survival rate among all the groups (**Supplementary Fig. 3**). In addition, with cardiovascular mortality as the endpoint, the results were similar to those obtained for all-cause mortality.

### 3.3 Cox Proportional Hazard Analysis of Outcome

**Fig. 2.**
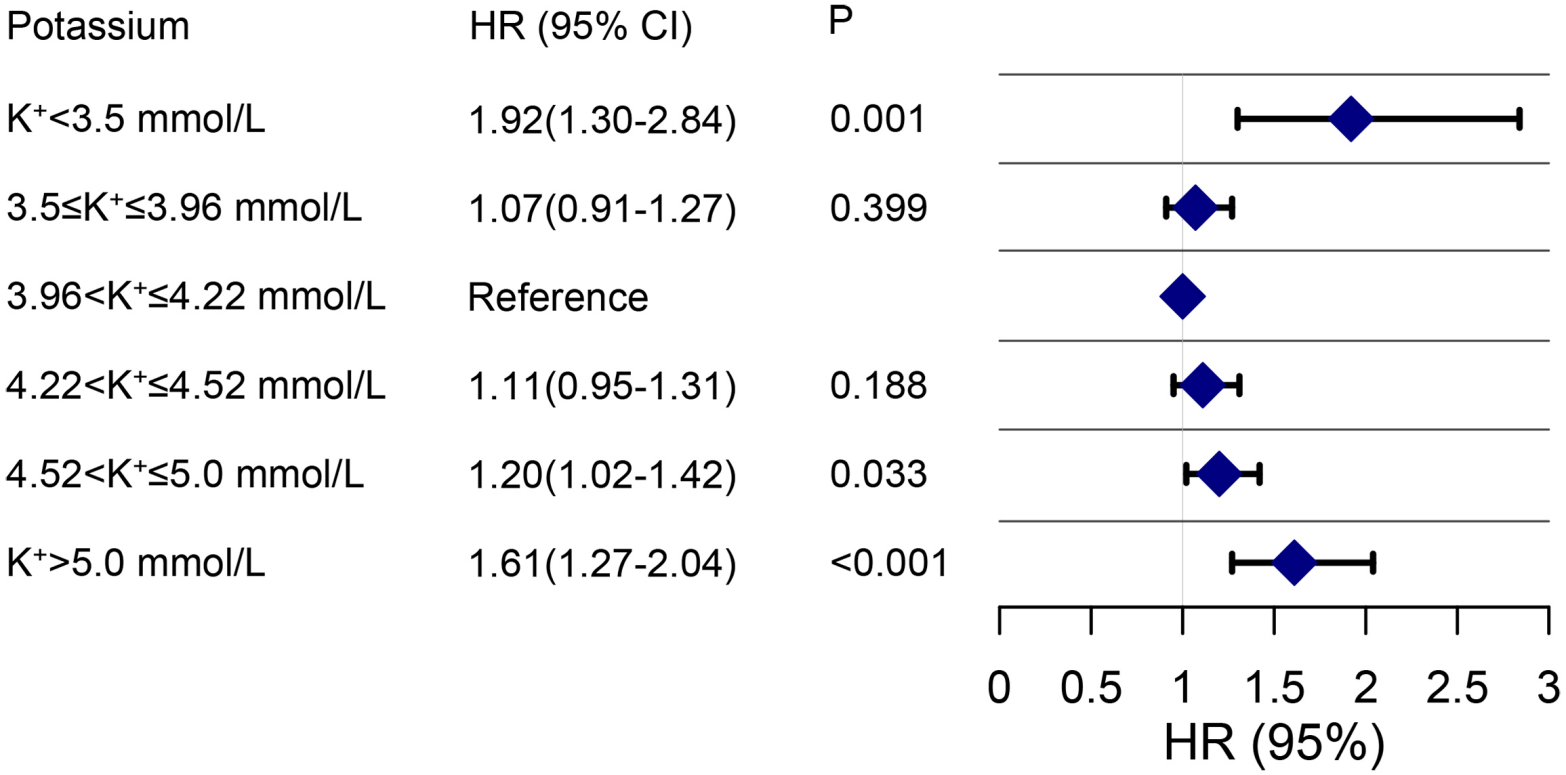
**Hazard ratios for maximal survival associated with serum potassium levels in heart failure patients**. The reference interval is the $K^{+}$ interval of 3.96–4.22 mmol/L. The adjusted variables were the same as those used for Model 3 in Table 2. HR, hazard ratio.

A univariate Cox regression model was utilized to identify significant variables that influence all-cause mortality. To evaluate the utility of serum $K^{+}$ levels in predicting all-cause mortality, we established three multivariate models. After multivariate adjustment, compared with the Q2 group, patients in the Q1 group did not demonstrate an increase in mortality at 90-days, 2-years, or maximal follow-up; however, patients in the Q4 group had an increased mortality rate. Table 2 shows the results of maximal follow-up assessments obtained using the multivariable-adjusted Cox model analysis with the Q2 group as a reference. In the adjusted analysis of Model 3, mortality did not significantly increase in patients in the Q1 group (hazard ratio [HR] 1.12, 95% confidence interval [CI]: 0.95–1.32, *p* = 0.180) and Q3 group (HR 1.12, 95% CI: 0.95–1.31, *p* = 0.178) but significantly increased in patients in the Q4 group (HR 1.28, 95% CI: 1.09–1.49, *p* = 0.002). During the 90-day, 2-year, and maximal follow-up, individuals with hypokalemia and hyperkalemia showed a substantial increase in mortality. The normal range of 4.52 < $K^{+}$ ≤ 5.0 mmol/L (HR 1.20, 95% CI: 1.02–1.42, *p* = 0.033) indicated an elevated risk of all-cause mortality, as shown in Fig. 2.

**Table 2.** **Cox hazard analyses of all-cause mortality based on serum potassium quartiles**.

Parameters	Model 1		Model 2		Model 3	
	HR (95% CI)	*p* value	HR (95% CI)	*p* value	HR (95% CI)	*p* value
$K^{+}$ ≤3.96 mmol/L (Q1)	1.13 (0.96, 1.33)	0.150	1.15 (0.98, 1.36)	0.085	1.12 (0.95, 1.32)	0.180
3.96 < $K^{+}$ ≤ 4.22 mmol/L (Q2)	1 (Reference)	-	1 (Reference)	-	1 (Reference)	-
4.22 < $K^{+}$ ≤ 4.52 mmol/L (Q3)	1.21 (1.03, 1.42)	0.019	1.21 (1.03, 1.41)	0.022	1.12 (0.95, 1.31)	0.178
$K^{+}$ >4.52 mmol/L (Q4)	1.47 (1.26, 1.72)	<0.001	1.50 (1.29, 1.75)	<0.001	1.28 (1.09, 1.49)	0.002

Model 1 was adjusted for sex and age; Model 2 was adjusted for Model 1 and hypertension, diabetes, coronary artery disease, digoxin, diuretics, angiotensin-converting enzyme inhibitors, beta-blockers, angiotensin II receptor blockers, and mineralocorticoid receptor antagonists; and Model 3 was adjusted for Model 2 and heart rate, body mass index, estimated glomerular filtration rate, N-terminal pro-B-type natriuretic peptide, systolic blood pressure, and New York Heart Association class. HR, hazard ratio; CI, confidence interval.

### 3.4 Restricted Cubic Spline Curve Analysis of Outcome

**Fig. 3.**
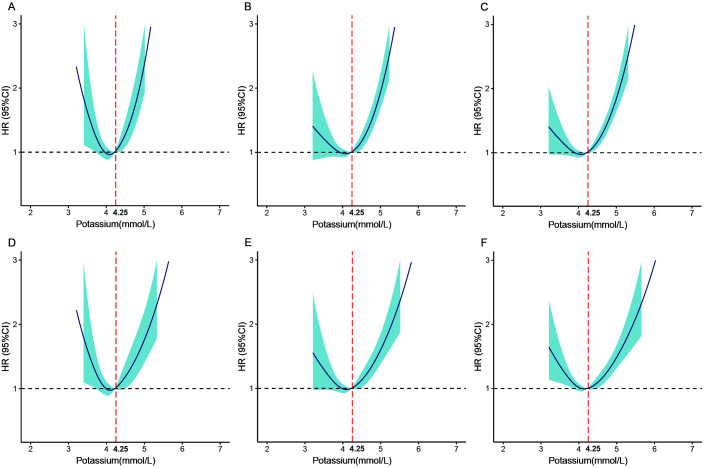
**Restricted cubic splines of the hazard ratios for all-cause mortality**. Unadjusted risk of mortality at 90-day (A), 2-year (B), and maximal (C) follow-up. The adjusted risk of mortality at 90-day (D), 2-year (E), and maximal (F) follow-up. Adjusted variables are the same as in Fig. 2. HR, hazard ratio.

The model was adjusted for demographic and clinical comorbidities and the use of relevant medications. The spline curve indicates that individuals with hypokalemia and those with hyperkalemia have an elevated risk of all-cause mortality. The spline curve also revealed that the lowest mortality risk was related to a serum $K^{+}$ level of 4.25 mmol/L. Fig. 3 depicts a J-shaped restricted cubic spline curve.

### 3.5 Subgroup Analysis

Using normal $K^{+}$ values of 3.5–5.0 mmol/L as a reference, with an adjusted HR of 1.53 (95% CI: 1.27–1.85, *p* < 0.001), an abnormal $K^{+}$ level (hypokalemia and hyperkalemia) was independently linked to an elevated risk of all-cause mortality. We found no significant interaction between abnormal $K^{+}$ levels and the relevant clinical subgroups or the use of baseline therapies (use of MRAs and ACEIs/ARBs). Hospitalized HF patients with abnormal $K^{+}$ levels are at an increased risk of death, regardless of whether they have diabetes or renal insufficiency (**Supplementary Fig. 4**).

## 4. Discussion

In this study, we observed that most hospitalized HF patients had $K^{+}$ levels before discharge that were within the 3.5–5.0 mmol/L range. After adjusting for potential confounding factors, hospitalized HF patients with hypokalemia and $K^{+}$ levels >4.52 mmol/L had higher 90-day, 2-year, and maximal follow-up all-cause mortality rate than those with the reference level of 3.96 < $K^{+}$ ≤ 4.22 mmol/L. Furthermore, our findings revealed that both hypokalemia and hyperkalemia were linked to elevated mortality in the short and long term. Moreover, the relationship between $K^{+}$ levels and mortality was depicted as a J-shaped curve, and the optimal $K^{+}$ range was narrower than normal serum $K^{+}$ levels.

Hypokalemia among hospitalized HF patients was linked to a higher mortality risk at the 90-day, 2-year, and maximal follow-up. In previous studies, hypokalemia was not associated with mortality at 3 months or 6 months after multivariate adjustment [15, 16]. After controlling for confounding factors, our research demonstrates that hypokalemia is an independent factor related to adverse outcomes in hospitalized HF patients. Various studies have indicated that hypokalemia is related to increased mortality risk in chronic HF patients [10, 17, 18, 19]. In HF and chronic kidney disease patients, a serum $K^{+}$ level of <4 mmol/L was related to higher mortality and incidence of hospitalization [19]. Furthermore, patients with chronic HF and hypokalemia still exhibit hypokalemia within 30 days, and their 90-day all-cause mortality risk is considerably greater than that of patients with $K^{+}$ levels in the 3.8–4.1 mmol/L range [20].

In hospitalized HF patients, hyperkalemia was linked to increased short- and long-term mortality. After adjusting all potentially confounding variables (including demographic and clinical features and medications), the relationship between $K^{+}$ levels >4.52 mmol/L and mortality still existed. The impact of renal function on serum potassium is very important and obvious and can regulate the level of serum $K^{+}$, and renal insufficiency can cause hyperkalemia. Hyperkalemia was substantially more frequent in individuals with chronic renal disease than in the general population, and cardiorenal syndrome can affect the prognosis of HF patients. In our study, patients in the Q4 group had lower eGFR levels, suggesting worse renal function, but after adjustment for eGFR, the association between the Q4 group and all-cause mortality remained significant. Patients in the Q4 group were older, had higher NT-proBNP, uric acid, high-sensitivity C-reactive protein, and had more cardiovascular comorbidities (hypertension, coronary artery disease, and lower renal function and hemoglobin levels). These factors may have influenced the analysis, and there may be confounding bias. In addition, comorbidities or severity of diseases may partly explain the worse prognosis of patients in the Q4 group, but the association persisted after these comorbidities were adjusted for. ACE inhibitors, ARBs, and MRAs are commonly used drugs for patients with HF that can increase serum $K^{+}$ levels and are common causes of hyperkalemia in patients [21]. Hyperkalemia is fairly common and frequently results in discontinuation of MRA therapy or dose reduction [22]. Previous studies have shown that using MRAs with careful monitoring of $K^{+}$ and creatinine levels is related to reduced hypokalemia and improved HF patient survival even when $K^{+}$ levels exceed 5.5 mmol/L [23, 24]. However, in our study, the proportions of patients in the Q1 and Q4 groups who used MRAs were similar. The proportion of patients using ACEIs or ARBs was higher in the Q4 group, yet there was no statistically significant difference in comparison with the other groups. In summary, our findings demonstrate that hyperkalemia may be a risk marker of disease severity and an independent factor associated with poor outcomes in HF patients.

Our research has several limitations. First, because the study was observational, we could not entirely rule out the effect of residual confounding factors. The cause and duration of HF were not considered. The research cohort was recruited from a single center, and the findings may not be generalizable to other populations; thus, a multicenter study is required. Second, as we did not investigate the dynamics of serum $K^{+}$ levels, we could not assess their influence on mortality. In addition, the combined use of $K^{+}$ supplements and different diuretics may affect serum potassium differently. Loop diuretic dosage may also be important information, and diet, drugs, or renal function often influence $K^{+}$ levels. The use of a single serum $K^{+}$ level to explore the connection between $K^{+}$ levels and long-term prognosis has limitations. Third, the association between changes in serum $K^{+}$ levels during hospitalization and outcome was not explored. Last, the link between abnormal $K^{+}$ levels and fatal arrhythmias or sudden cardiac death remains unclear.

## 5. Conclusions

This research revealed a J-shaped connection between $K^{+}$ levels and all-cause mortality in hospitalized HF patients, with both hypokalemia and hyperkalemia linked to increased mortality. Likewise, patients in the Q4 group had substantially greater short-term as well as long-term all-cause mortality than those in the Q2 group, suggesting that a $K^{+}$ range narrower than the normal range should be targeted in hospitalized HF patients.

## Data Availability

Data supporting the findings of this study are available from the corresponding author upon reasonable request within 1 year of publication of this article.

## Supplementary Material

Supplementary material associated with this article can be found, in the online version, at https://doi.org/10.31083/j.rcm2408228.
