# Supplementary material for: Serum Potassium Levels and Mortality in Hospitalized Heart Failure Patients
Source: Rev Cardiovasc Med. 2023 Aug 9;24(8):228. doi: 10.31083/j.rcm2408228 (PMC11266832; doi:10.31083/j.rcm2408228)
Supplement: Supplementary file 1 [file 2153-8174-24-8-228-s1.docx]

**Supplementary Material**


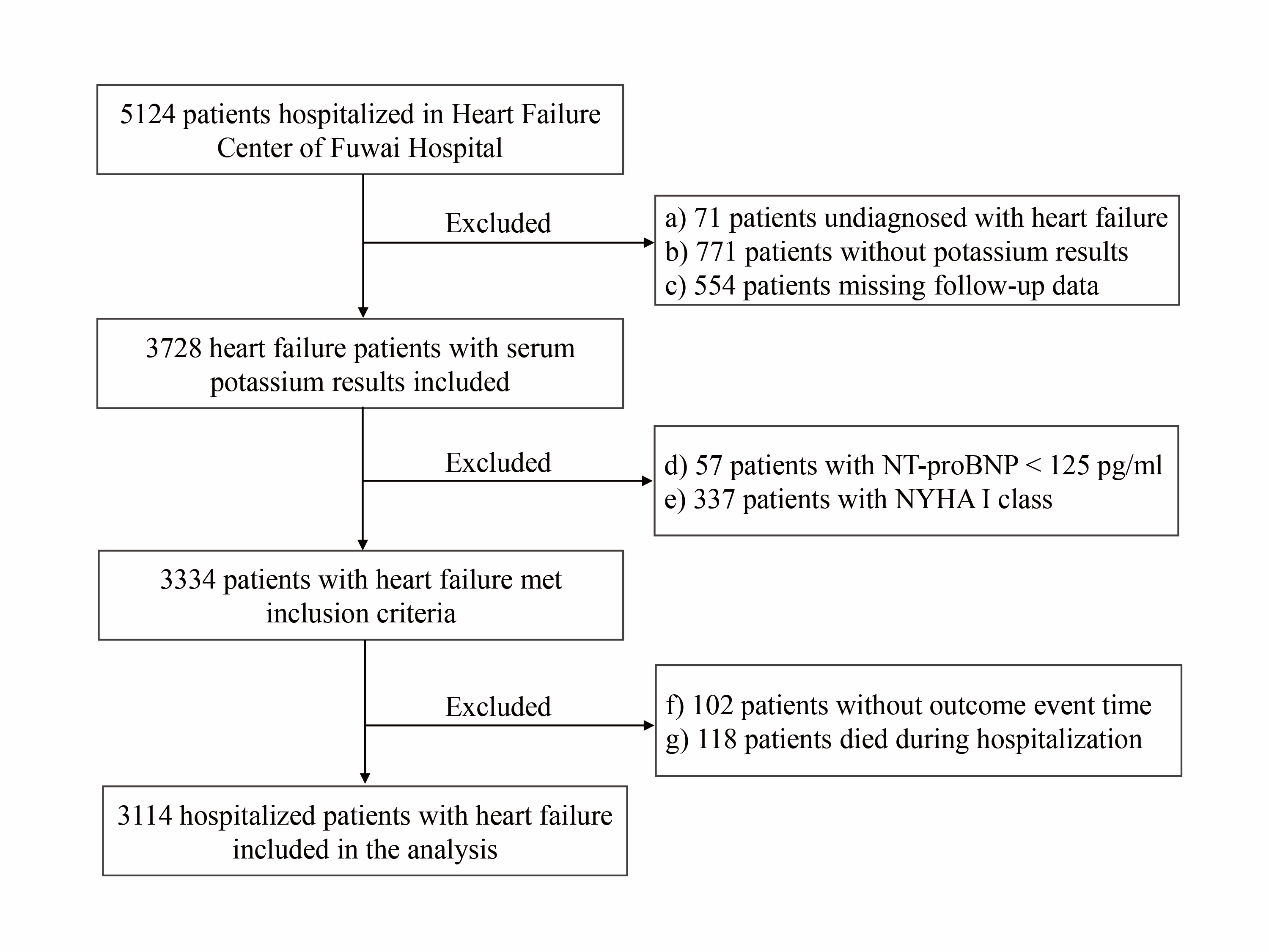


**Supplementary Fig. 1: Study flowchart.** NT-proBNP, N-terminal pro-B-type natriuretic peptide; NYHA, New York Heart Association.


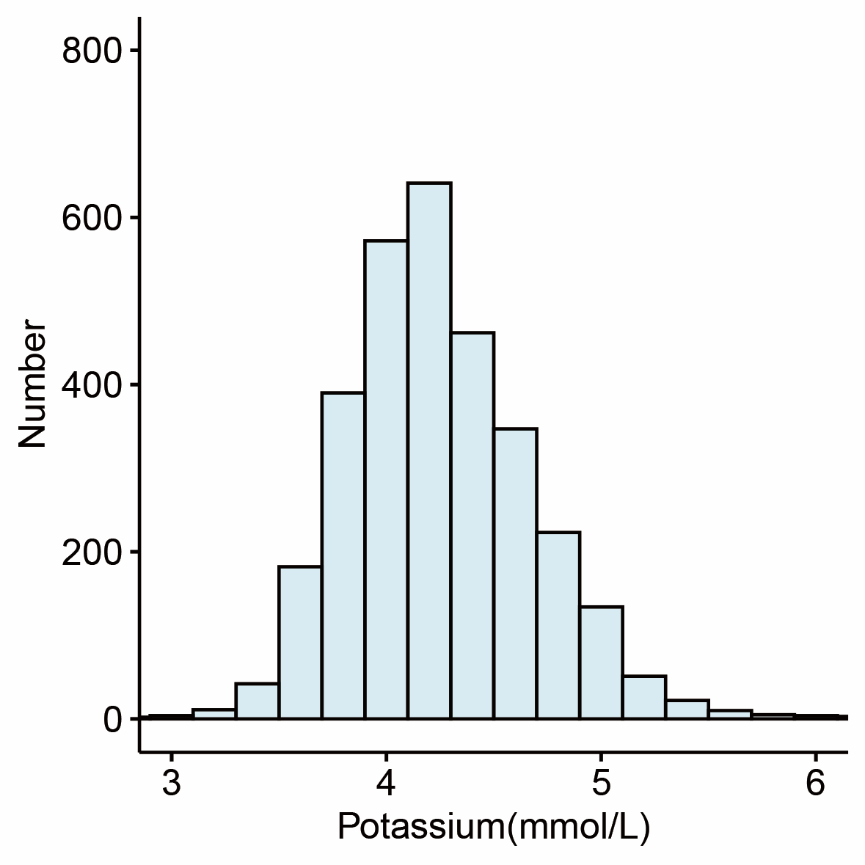


**Supplementary Fig. 2: Distribution of baseline potassium levels in the study population.**


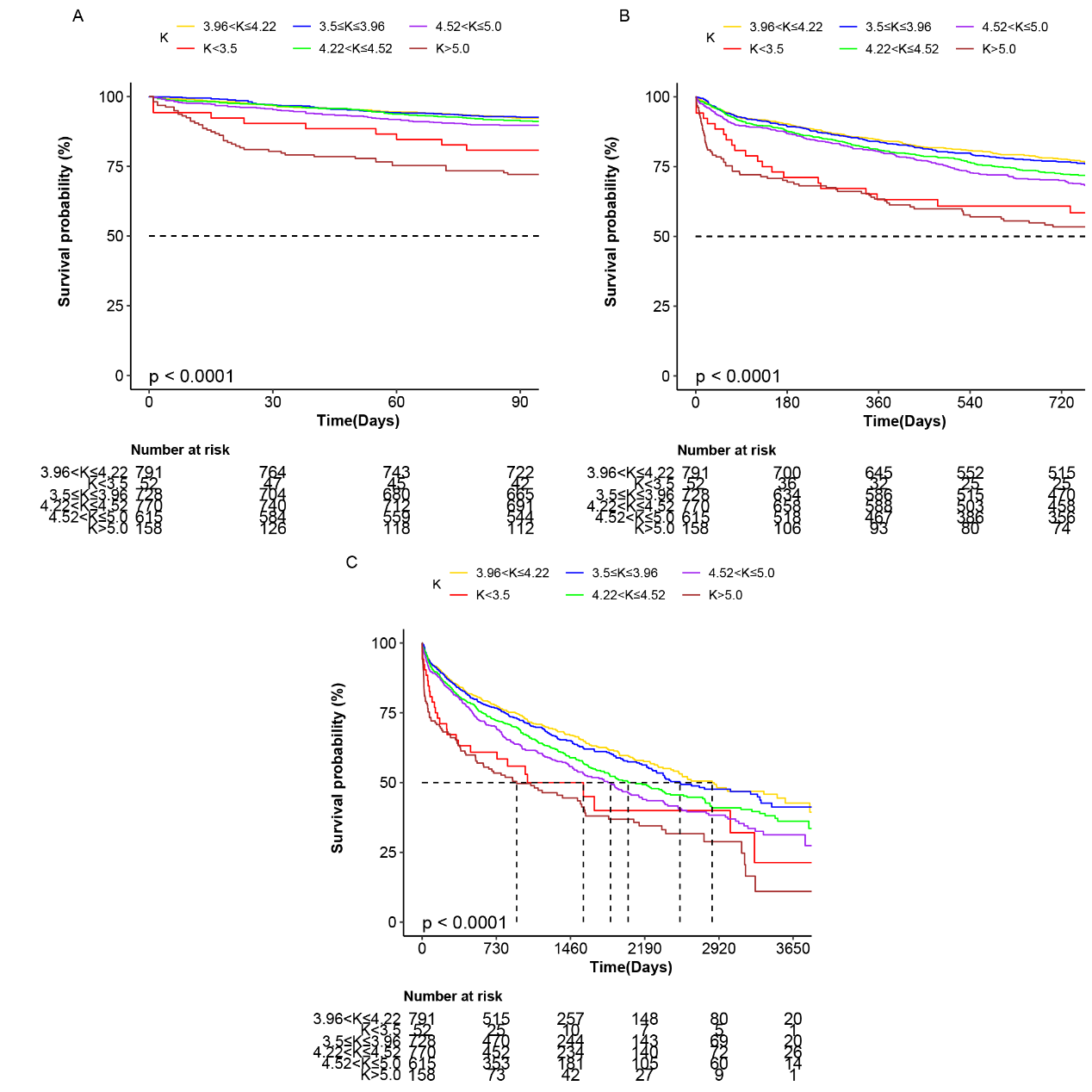


**Supplementary Fig. 3: Kaplan‒Meier analysis of the probability of all-cause mortality among patients with different potassium levels.** Ninety-day (A), 2-year (B), and maximal follow-up survival (C) for six groups: red, K^+^<3.5 mmol/L; blue, 3.5≤K^+^≤3.96 mmol/L; yellow, 3.96<K^+^≤4.22 mmol/L; green, 4.22<K^+^≤4.52 mmol/L; purple, 4.52<K^+^≤5.0 mmol/L; and brown, K^+^>5.0 mmol/L.


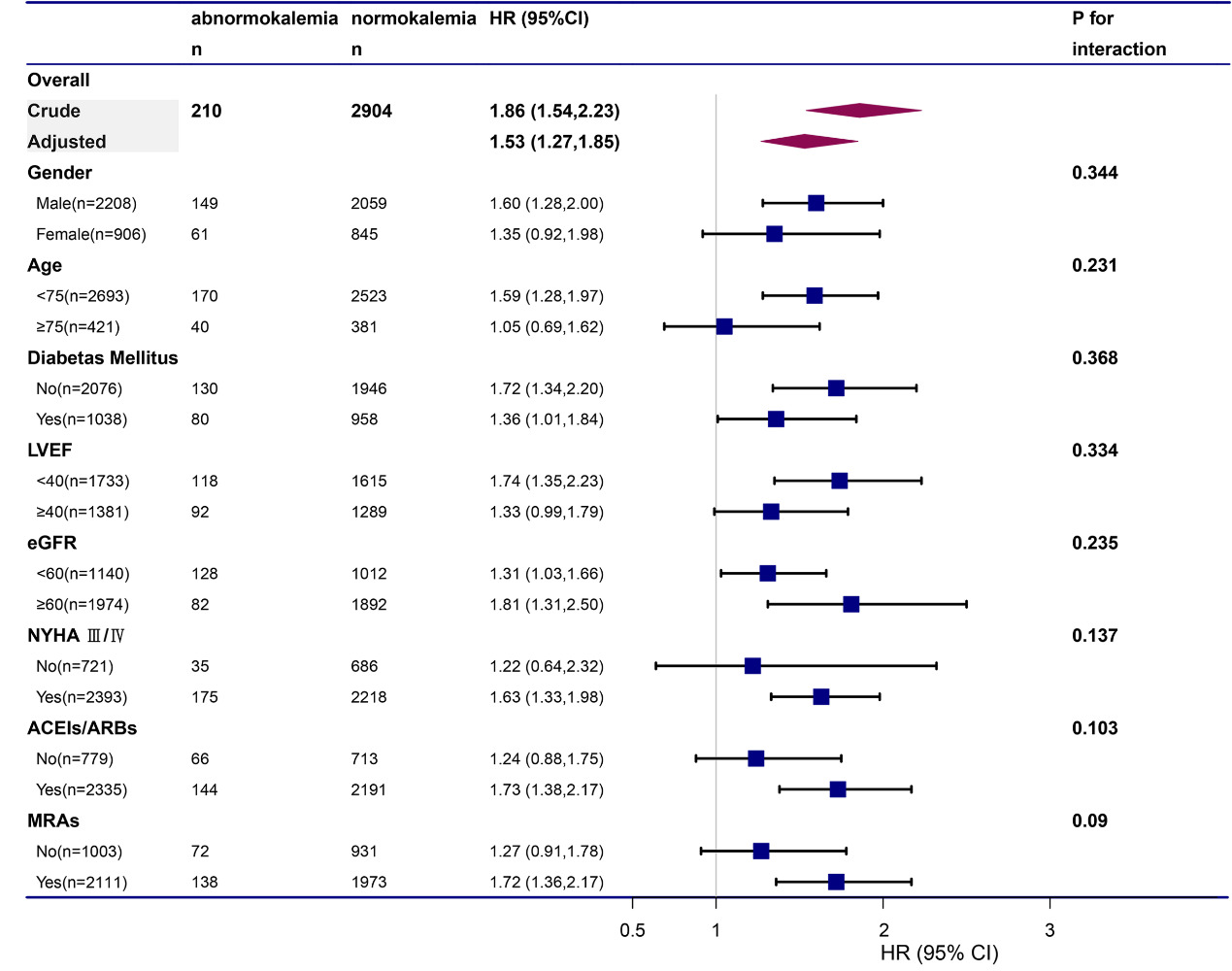


**Supplementary Fig. 4: Subgroup analysis of serum potassium levels and all-cause mortality.** ACEIs, angiotensin-converting enzyme inhibitors; ARBs, angiotensin II receptor blockers; CI, confidence interval; HR, hazard ratio; LVEF, left ventricular ejection fraction; MRAs, mineralocorticoid receptor antagonists; NYHA, New York Heart Association. Adjusted variables are the same as in Fig. 2.
